# The utility of pharmacological and radiological interventions to optimize diagnostic information from PET/CT

**DOI:** 10.1186/s40644-020-00344-9

**Published:** 2020-09-22

**Authors:** David Dudoignon, David A. Pattison, Damien Legallois, Rodney J. Hicks, Nicolas Aide

**Affiliations:** 1 The Department of Nuclear Medicine, University Hospital, Caen, France; 2 Department of Nuclear Medicine & Specialised PET Services, Royal Brisbane and Women’s Hospital, Herston, Australia; 3 School of Medicine, University of Queensland, Brisbane, Australia; 4 The Department of Cardiology, University Hospital, Caen, France; 5 The Department of Molecular Imaging and Therapeutic Nuclear Medicine, the Peter MacCallum Cancer Institute, Melbourne, Australia; 6 The Sir Peter MacCallum Department of Oncology, the University of Melbourne, Parkville, Australia; 7 INSERM ANTICIPE, Normandie University, Caen, France

**Keywords:** Positron emission tomography, False-negative, Intervention, Methodology, Protocol

## Abstract

**Background:**

Positron Emission Tomography with Computed Tomography (PET/CT) is widely used in the assessment of many diseases, particularly including cancer. However, many factors can affect image quality and diagnostic performance of PET scans using FDG or other PET probes.

**Main body:**

The aim of this pictorial essay is to review PET/CT protocols that can be useful to overcome these confounding factors in routine clinical situations, with a particular focus on pharmacological interventions and problem-oriented CT acquisition protocols.

**Conclusion:**

Imaging protocols and representative cases will be discussed, in addition to potential contraindications and precautions to be taken.

## Background

There are many factors that can affect image quality and diagnostic performance of PET/CT examinations using FDG or other PET probes. We will review pharmacological interventions (including dosage and contraindications) and use of problem-oriented radiological imaging protocols designed to optimize diagnostic information and improve the overall accuracy of PET/CT in routine clinical practice. Herein, we present representative cases with and without drug intervention to illustrate how useful these simple protocols are.

Illustrative cases are presented with a thresholding proposed by the Peter Mac Callum Cancer Centre (PMCC) team for FDG studies with the use the “rainbow” colour scale that has low activity regions displayed in the blue-green range and higher intensity regions in the orange-red spectrum. With this colour scale, the SUV threshold should be adjusted so that the liver appears blue with flecks of green. This corresponds to an upper SUV window threshold of approximately 9, except in bariatric patients where the upper SUV threshold is typically 12 [1]. While the same colour scale is used for other tracers, the SUV range and reference organ may vary. For example, for somatostatin receptor imaging using ^68^Ga-DOTA-octrotate an upper SUV threshold of 30 is typically used while for PSMA ligands the corresponding upper threshold at PMCC is 15. Lower thresholds may increase sensitivity, but this is generally at the expense of specificity, whereas higher thresholds can provide greater appreciation of heterogeneity between tumour sites but lead to faint lesions becoming inapparent.

While there has been a strong professional and industry focus on harmonisation of CT windowing for various tissues, thresholding of PET images remains largely idiosyncratic and non-standardised even sometimes within departments according to individual reader preferences. At the very least, the authors recommend that each department has a consistent method of displaying images across readers and through time in order to facilitate comparative studies.

The cases presented represent examples from three PET facilities primarily staffed by nuclear medicine physicians but including dual-trained radiologists. The PMCC facility operates within a combined Cancer Imaging Department with CT protocols developed collaboratively with the CT modality leads. Where variations in protocols exist, these are discussed.

## Diabetic patient: correction of hyperglycemia with insulin (Fig. 1)

Hyperglycemia directly competes with FDG for uptake in normal tissues, particularly the brain, and also in tumour. This alters the tumour-to-background ratio, potentially decreasing the sensitivity of the PET scan for detection of malignancy [2, 3], especially for lesions with low intrinsic metabolic activity [4]. It also compromises quantitative assessment of measures including the standardized uptake value (SUV), limiting the utility of such measures for therapeutic monitoring.

**Fig. 1:**
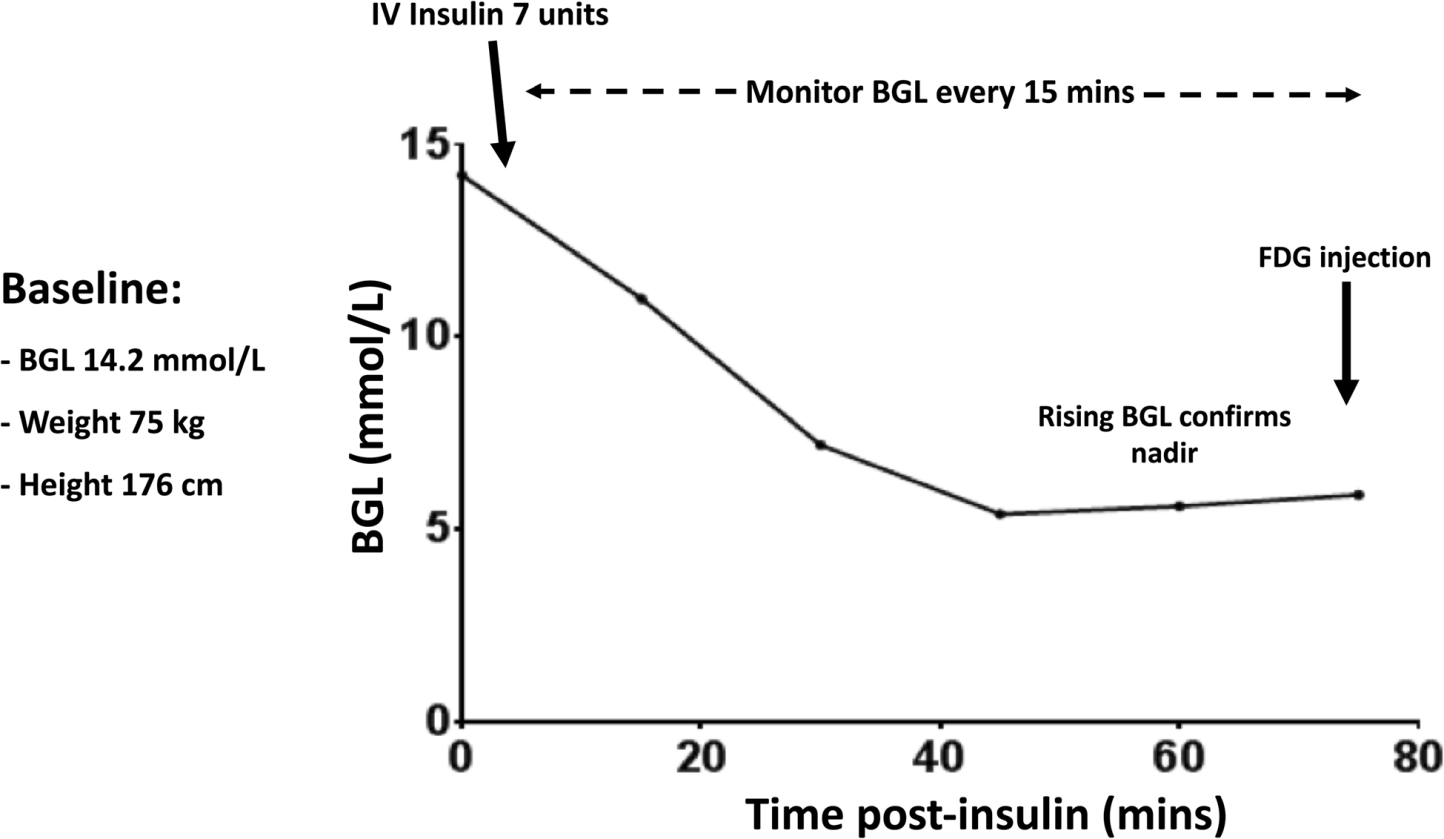
Schematic outlining the procedure for use of insulin calculator for correction of hyperglycemia with insulin

It should be noted that even under fasting conditions, normal individuals have a basal insulin level, but this is lacking in type 1 diabetes mellitus (DM) and insufficient to overcome insulin resistance in insulin-dependent type II DM. Therefore, completely withholding insulin is such patients is inappropriate. Nevertheless, careful management to avoid acute alterations of glucose homeostasis are required to avoid impacting the biodistribution of FDG, particularly under the influence of the insulin-dependent glucose transporter GLUT-4, which is preferentially expressed in cardiac and skeletal muscle. Accordingly, management of type 1 DM or insulin-dependent type II DM should avoid FDG being injected within 4 h of subcutaneous administration of rapid-acting insulin. It is, however, preferable for diabetic treatment to be maintained to avoid hyperglycaemia. This can be achieved by;

- Schedule FDG PET examination in the early morning in patients having received intermediate-acting or long-lasting insulin in the evening
- Or schedule FDG PET examination in the early morning in patients treated with continuous insulin infusion, the insulin pump being turned-off 1–2 h prior to FDG injection and administering FDG when blood sugar levels begin to rise, indicating loss of exogenous insulin effect. Avoid corrective bolus insulin 4–6 h of FDG injection.

However, for patients who present with hyperglycaemia despite these recommendations, the threshold of blood glucose level (BGL) that irrevocably impairs diagnostic performance is currently unclear and strategies for cancelling imaging or intervening to correct hyperglycemia vary between organisations.

The consensus clinical guidelines of major societies recommend rescheduling patients with hyperglycaemia above specified thresholds (Society for Nuclear Medicine [SNM] > 150–200 mg/dL [5] [8.3–11.1 mmol/L], National Cancer Institute [NCI] > 200 mg/dL [6] [11.1 mmol/L]; European Association for Nuclear Medicine [EANM] > 11 mmol/L [about 200 mg/ dL] for clinical studies or > 7.0–8.3 mmol/L [126–150 mg/dL] for clinical trials [7]; International Atomic Energy Agency [IAEA] > 200 mg/dL [8] [11.1 mmol/L]), or consideration of insulin administration for correction of hyperglycaemia in such circumstances.

To lower blood glucose levels under these pre-defined thresholds, different PET scan preparation protocols have proposed the intravenous administration of rapid acting insulin. In a recent study of Pattison et al. [9], a personalized insulin calculator protocol effectively lowered BGL to the target range, resulted in significantly fewer hypoglycemic events and reduced median time between insulin and FDG injection compared to pre-existing empiric protocols [10]. Inputs to the web-based calculator include patient BGL (mmol/L), weight (kg) and height (cm), which is freely available at https://www.petermac.org/services/diagnosis-investigations/positron-emission-tomography-pet/fdg-pet-insulin-calculator. This protocol uses intravenous short-acting insulin with close monitoring of the BSL until it reaches a nadir, typically within 1 h, and begins to rise again, indicating that the effects of exogenous insulin have dissipated. With this protocol, the majority of patients (88.3%) achieved BGL < 10.0 mmol/L, there was minimal risk of hypoglycaemic events (0.7%) and high-quality PET images were obtained. It should be stressed again that giving insulin contemporaneously with FDG is contraindicated due to its ability to drive FDG into cardiac and skeletal muscle under the influence of the insulin-dependent glucose transporter, GLUT-4. Conversely, if symptomatic hypoglycemia is induced, whether requiring glucose supplementation or not, scanning should be cancelled as this generally invokes a catecholamine response that may increase brown fat activation.

## Propranolol for prevention of brown fat uptake (Fig. 2)

Physiologic uptake of FDG in brown adipose tissue (BAT) of cancer patients may confound interpretation of PET scans. It occurs in half of paediatric oncology patients and is especially common in adolescents [11]. BAT uptake is particularly problematic as it occurs in common sites of metastatic cancer, such as the lateral neck, supraclavicular and mediastinal regions. Although careful correlation with CT images can exclude structural abnormalities associated with sites of uptake, residual nodes may be difficult to characterise and the altered biodistribution of FDG can impact semi-quantitative measures of uptake, including the standardised uptake value (SUV) of even lesions beyond the distribution of BAT and thereby potentially compromise therapeutic response assessment.

**Fig. 2:**
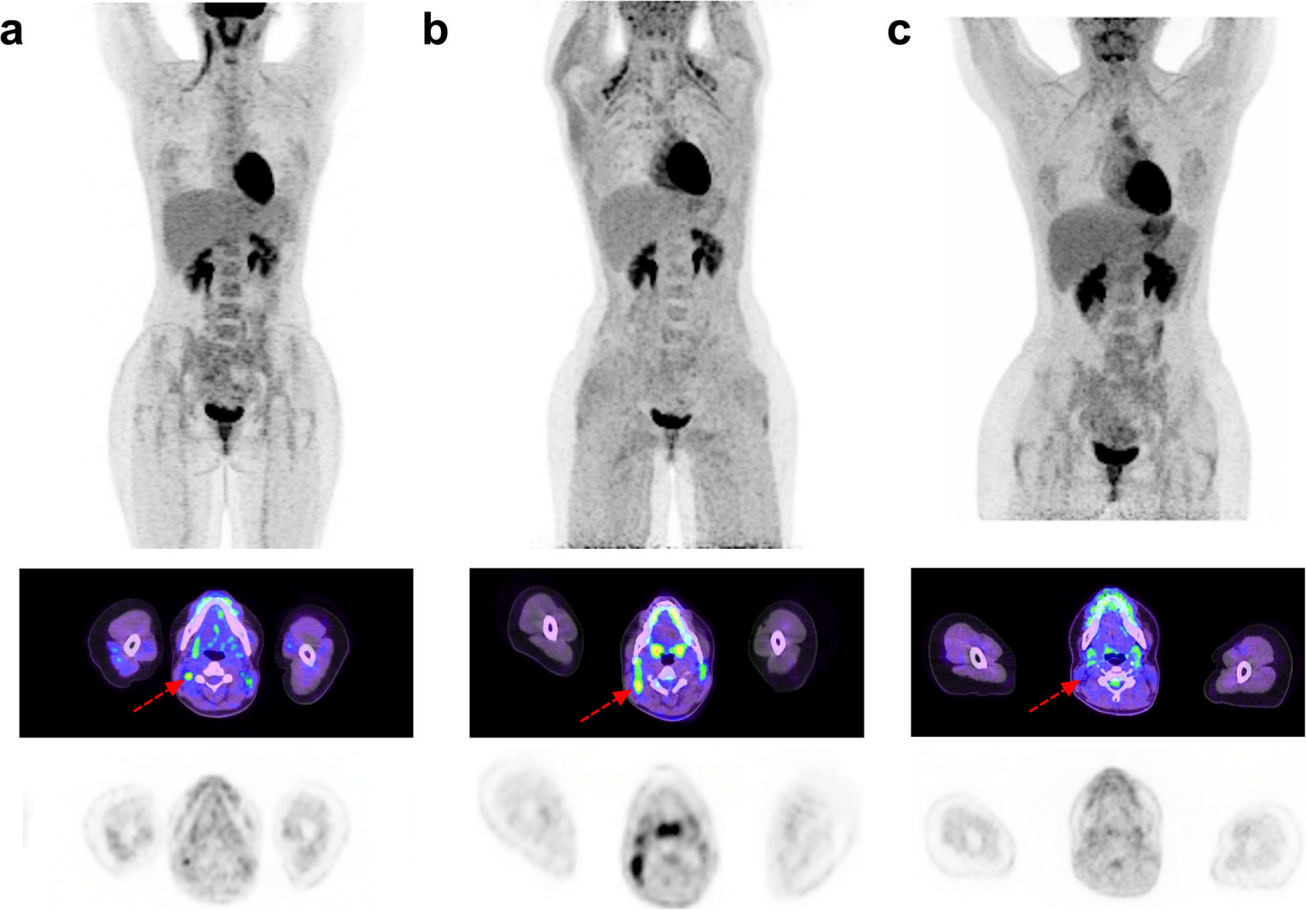
31-year-old female with Hodgkin Lymphoma. From top to bottom, Maximum intensity projection (MIP) PET, fused axial PET/CT and stand-alone PET images: **a** June 2018: Baseline PET showing stage 2 disease in the right neck. **b** January 2020: End-of-treatment PET considered non-diagnostic due to BAT activation in sites involved on baseline scan. **c** March 2020: follow-up PET examination. The patient received 40 mg of propranolol 1H prior to FDG injection. BAT activation is no longer visible and the patient is considered in complete metabolic response (Deauville score 2) with mediastinal uptake interpreted as thymic hyperplasia

BAT regulates body temperature by inducing thermogenesis. Although previously thought to be primarily an issue in cold climates, it can occur even in temperate or tropical climates when patients come into an artificially-cooled environment. Therefore, the temperature of uptake rooms should be regulated to avoid cold stress. Warm blankets may assist if air-conditioning cannot be adequately regulated.

Since BAT is innervated by the sympathetic nervous system, stress or elevated catecholamines levels (for example in patients with functional pheochromocytoma or paraganglioma), may be associated with elevated BAT uptake. Accordingly, beta blockers such as propranolol have shown efficacy in reducing BAT uptake [12]. Low blood sugars can be seen in infants and even in older children who have been fasting, as required prior to FDG PET/CT, and can induce secondary catecholamine-induced BAT activation.

Propranolol has been used in paediatrics to treat a variety of conditions. Although generally safe, it can cause bradycardia, hypotension and hypoglycemia. There are only few contraindications, including chronic obstructive pulmonary disease (COPD) and asthma, and various cardiovascular conditions including uncontrolled heart failure, bradycardia, hypotension, high grade AV block, sinus disease, Prinzmetal angina and Raynaud disease. It should also be avoided in patients with known or suspected pheochromocytoma / paraganglioma (e.g. assessment of adrenal / paravertebral lesion with elevated BAT uptake) unless adequately alpha-blocked due to a risk of hypertensive crisis from unopposed alpha-adrenergic activation [13]. For adults, the recommended dosage is 20–40 mg Propranolol 1 h before the PET examination, as used in the study of George et al. [12]. In children, 5-10 mg can be prescribed in consultation with a paediatrician.

It is noteworthy that some groups use benzodiazepines to reduce BAT uptake, though efficacy of benzodiazepines in this setting remains questionable. In a preclinical study Tatsumi et al. [14] found no efficacy of diazepam, as opposed to propranolol, and a randomized trial evaluating the effect of diazepam (5 mg orally) on BAT found no difference with the placebo group.

## Improvement of cardiac imaging with special diet and heparin (Fig. 3)

Adequate suppression of cardiac glucose metabolism increases the interpretability and diagnostic reliability of 18F-FDG PET/CT studies performed to detect cardiac inflammation and infection or, in rare cases, of primary or secondary malignant involvement of the heart or pericardium.

**Fig. 3:**
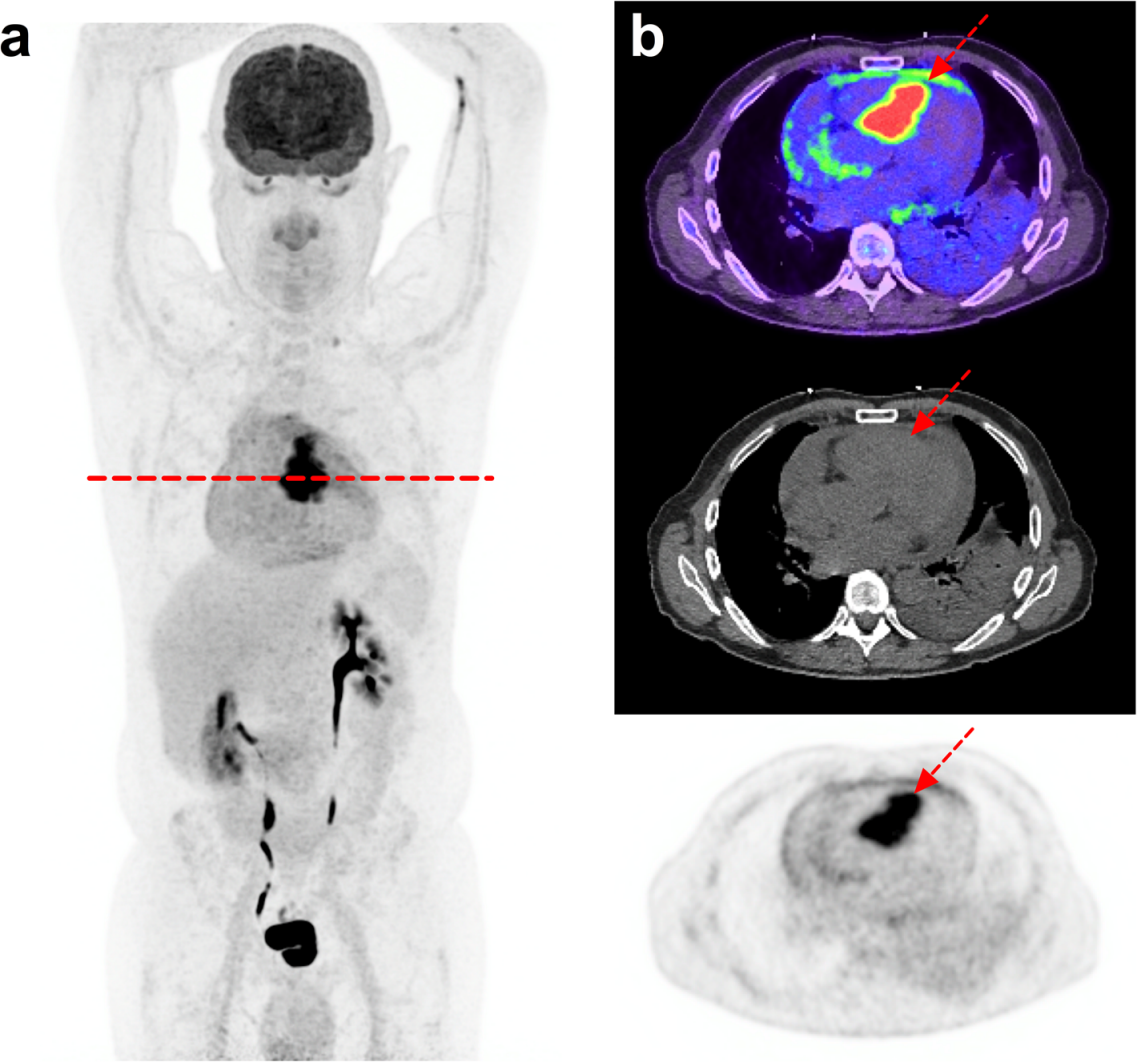
69-year-old male referred for suspicion of myocardial metastasis from melanoma. From left to right, MIP PET (**a**) and axial fused PET/CT, CT and PET images (**b**): Prolonged fasting (12 h), carbohydrate-restricted diets, fatty meals, and heparin loading (50 UI/Kg 15 min prior to FDG injection) allowed suppression of physiological myocardial uptake and clear visualization of a septal metastasis and metastatic pericardial effusion

There are no standardized guidelines, though prolonged fasting (beyond 24 h), carbohydrate-restricted diets, fatty meals, and heparin loading all have been proposed.

At the Caen University Hospital, the protocol involves high-fat, low-carbohydrate diet on the day prior to scanning followed by prolonged fasting over 12 h. This is followed by unfractionated heparin (50 UI/kg) being injected 15 min before the FDG injection. The aim of this protocol is to decrease basal insulin and blood glucose levels and to increase blood free fatty acid (FFA) levels, which shift myocardial energy consumption away from glucose toward FFA. At the Royal Brisbane & Women’s Hospital, an even more aggressive dietary regimen is instituted to invoke ketogenesis. This involves a high-fat diet for 48 h (incorporating prolonged fast for final 18 h), then heparin in patients without contraindications. Ketone monitoring (capillary and urine) has also been performed to assess the adequacy of dietary preparation for cardiac sarcoid imaging, and if not elevated, prompt more detailed assessment of dietary adherence [15]. Type 4 cellular glucose transporter (GLUT4) mediates the uptake of FDG in the myocardium whereas it is GLUT1 or GLUT3 that mediate the increased glucose consumption of inflammatory cells or tumours.

Unfractionated heparin significantly improves image contrast, as demonstrated by Scholtens et al. [16] by using a single-dose heparin pre-administration in addition to a low-carbohydrate diet. In their study, this protocol outperformed a low-carbohydrate diet alone in adequately suppressing cardiac glucose metabolism (88% vs 54%, *p* < 0.0001). The main side-effect is bleeding risk and the contraindications include children under 3 years of age, severe thrombocytopenia, heparin-induced thrombocytopenia (HIT) hypersensitivity or any situation which may cause bleeding. The bleeding risk is, however, very low if used properly as a single dose. For example, there was no bleeding in the study from Scholtens et al. [16] in which 150 patients were included.

Heparin should be avoided in patients who are already receiving anticoagulant therapy or have a history of bleeding disorders. When using this protocol for PET tumour imaging, for instance for detection of myocardial metastases, attention should be paid to tumours at risk of bleeding and brain metastases. Heparin-induced thrombocytopenia from unfractionated heparin was calculated at 2.6% in a meta-analysis on thromboprophylaxis by Martel et al. [17] but the duration of heparin therapy (range 6-30d) was far longer.

## Gastric distension with scopolamine for staging/restaging of gastric malignancy (Fig.4)

Physiologic smooth muscle uptake in the empty stomach of fasting patients often hampers the visualization of focal or diffuse gastric lesions by increasing apparent background activity.

**Fig. 4:**
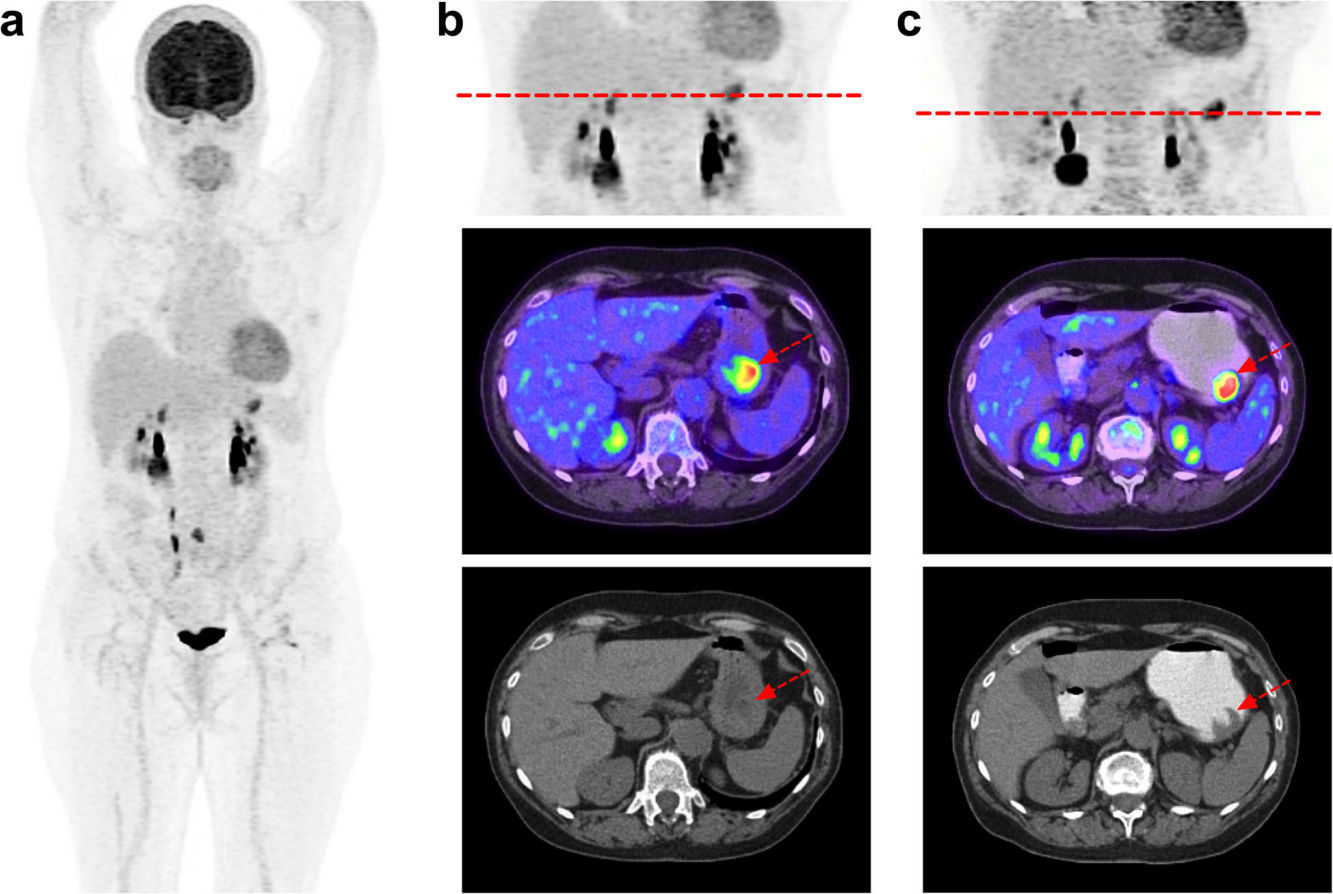
63-year-old woman, referred for assessment of metastatic melanoma (brain, lungs, lymph nodes) after 17 cycles of second-line Pembrolizumab. Her medical record reported anaemia. From left to right, MIP body PET and from top to bottom, abdominal MIP, PET/CT fused and CT axial slices: **a** and **b** Images 60 min after FDG injection. **c** delayed images acquired 15 min after 20 mg scopolamine intravenous infusion and oral contrast agent (500 cc) that permitted to dilate and fulfil the stomach showing an ulcered gastric metastasis, confirmed at endoscopy and likely to be the cause of the reported anaemia

Based on the study from Le Roux et al. [18], the protocol that we propose is a 20 mg scopolamine infusion 20 min before the acquisition associated with oral contrast agent solution ingested just before the image acquisition (500 ml of water +/− 10 mL of Gastrografine). Scopolamine is a choline receptor blocking that relaxes smooth muscle and reduces gastro-intestinal motility. Oral contrast agent induces full gastro-intestinal expansion and maintains tension in the wall. It also provides a good contrast in CT between the low density of gastric cavity and the moderate density of the gastric wall [19]. Several other protocols have been proposed [20–22], using regional gastric images a few minutes after only water oral, intake (> 300 mL), after the whole-body scan has been performed.

Of note, because of their effect on attenuation correction CT maps, oral contrast agent can cause an increased SUV in the gastro-intestinal tract and thus causes artifacts. Therefore, we recommend keeping the same imaging protocol for follow-up imaging, for instance in the neo-adjuvant setting, so that variation in SUV between baseline and post-chemotherapy scans is not hampered by contrast media.

Scopolamine (Scoburen/Buscopan) is easy to use and there are relatively few contraindications. These include known hypersensitivity, risk of acute angle-closure glaucoma, a past history of urinary retention or significant prostatism in men and this treatment is not recommended during the third trimester of pregnancy or when women are breast feeding.

## Forced diuresis using diuretics for bladder tumour imaging (Fig. 5)

FDG has been used with limited success in the past in primary diagnosis and locoregional staging of urinary bladder cancer or other urothelial tumours, mainly because of the pharmacokinetics of renal excretion.

**Fig. 5:**
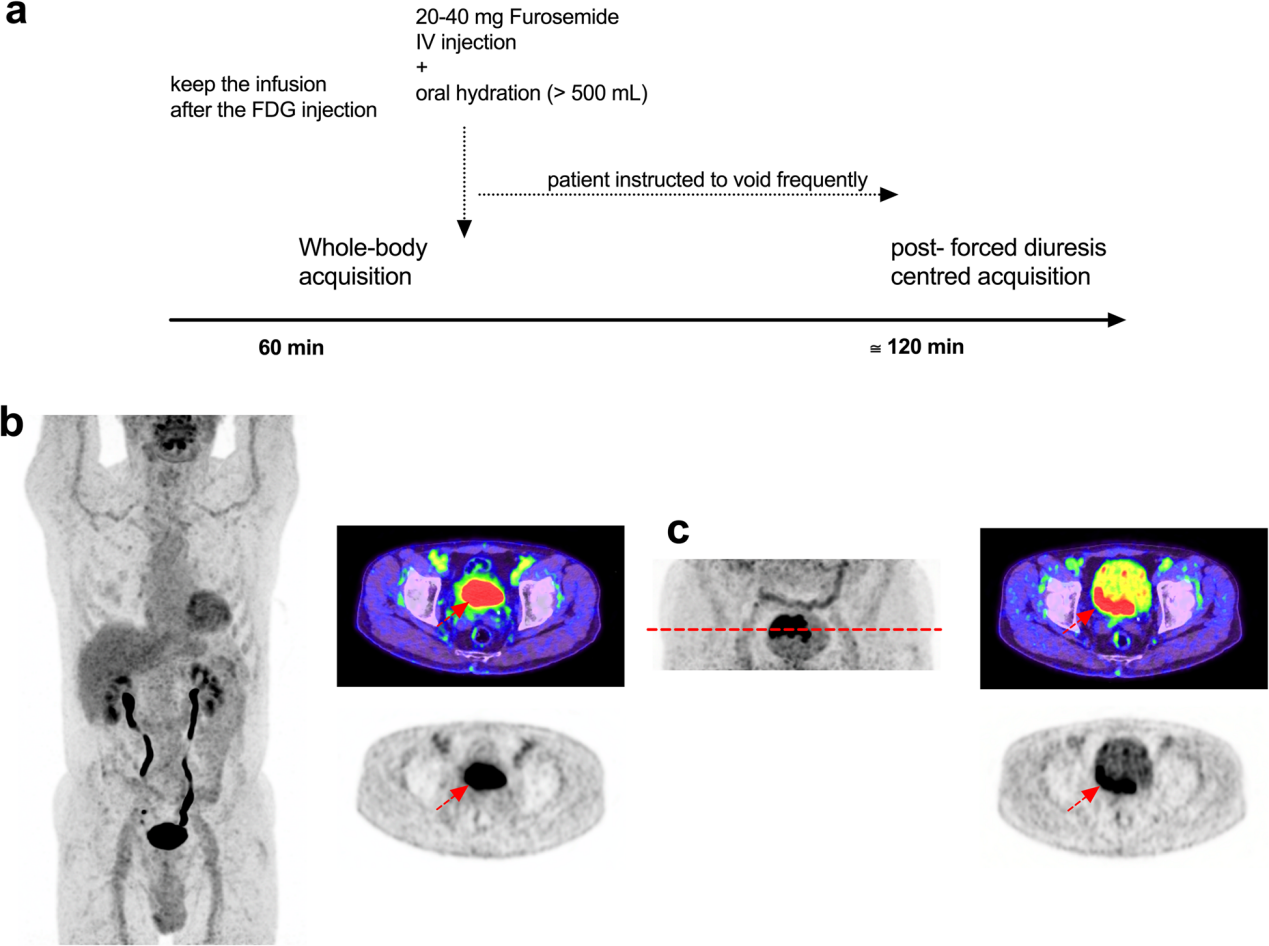
73-year-old man, referred for staging after bladder polyposis resection. **a** Schematic outlining of the procedure for use of diuretics. **b** MIP and axial fused PET/CT and PET images through the bladder at 1 h after FDG injection. **c** Localised MIP of the pelvis and axial fused PET/CT and PET images 30 min after 20 mg furosemide intravenous infusion, acquired 120 min after FDG injection. Radioactive urine is eliminated from the bladder and enabling visualisation of the posterior wall tumour

Indeed, pooling of urinary radiotracer in the ureters, bladder or urachus can obscure urinary tract lesions even when they have high metabolic activity, which is typically the case for transitional cell carcinoma (TCC). While FDG PET/CT remains useful for detecting nodal and distant spread of TCC, without alteration of scanning protocols the detection of primary disease is compromised.

This is optimally achieved by diluting activity in the bladder by using a diuretic drug such as furosemide (Lasilix/Lasix) in addition to water oral intake (> 500 mL). Diuretics can be used even in patients with kidney failure but at increased doses [23–25]. Delayed imaging in excess of 120 min also enhances urinary clearance, particularly if combined with prior diuretic administration. Use of radiographic contrast approximately 10–15 min prior to delayed imaging may also help delineation of focal lesions and for detecting exophytic bladder lesions.

The contraindications are kidney obstruction, acute kidney injury, hepatic encephalopathy, dehydration, severe hypokalaemia, severe hyponatraemia, severe kidney failure (eGFR < 30 ml/min), hepatitis and liver failure.

## Use of CT Urogram protocols for detection of nodes adjacent to the ureters (Fig. 6)

Focal urinary collection of activity in the ureters of radiotracers that are renally-excreted can be mistaken for nodal disease. This is particularly common at the pelvic brim and in the lateral pelvis where the ureters cross major vessels. Primary genito-urinary tumours including prostate, endometrial and cervical cancers often involve pelvic, iliac and retroperitoneal nodes in close proximity to the ureters as well as directly involving these structures. With the increasing use of imaging of prostate cancer using prostate-specific membrane antigen (PSMA) agents, use of a delayed CT urogram protocol can assist in increasing the confidence with which nodal disease can be diagnosed or excluded [26]. This involves administration of approximately 50-100 ml of radiographic contrast approximately 10–15 min prior to PET/CT acquisition. This is particularly beneficial for Ga-68 agents, due to the limitations for delayed, post-diuretic imaging imposed by the relatively short half-life of this radionuclide [27]. Additionally, focal pooling of urine can still be present even after forced diuresis. This protocol might also be useful for evaluating pelvic malignancies with FDG, particularly cervical cancer which can invade the parametrium and thereby compromise ureteric drainage or the adjacent bladder as well as commonly involving pelvic, iliac and retroperitoneal nodes.

**Fig. 6:**
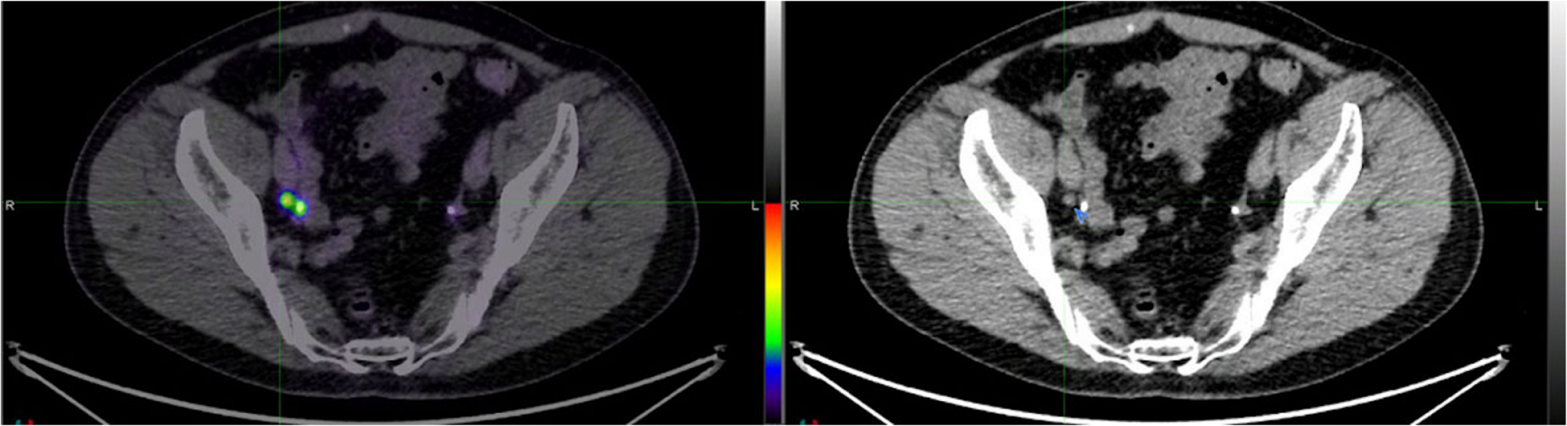
PSMA PET/CT imaging in this male with biochemical recurrence of prostate cancer demonstrates activity in the distal ureter, which is clearly identified by colocation with radiographic contrast on the fused image (left) whereas the small node lateral to this is also seen, consistent with small volume nodal metastasis

## Uterine cervical tumours (Fig. 7)

The non-medication intervention of use of a vaginal tampon can be proposed to women prior to the image acquisition. The tampon stretches the vagina and allows better delineation of the inferior margin of the uterine cervix. There is no specific contraindication but this intervention should be used with caution in symptomatic patients after radiotherapy or in old women with vaginal dryness, since this may cause pain and bleeding.

**Fig. 7:**
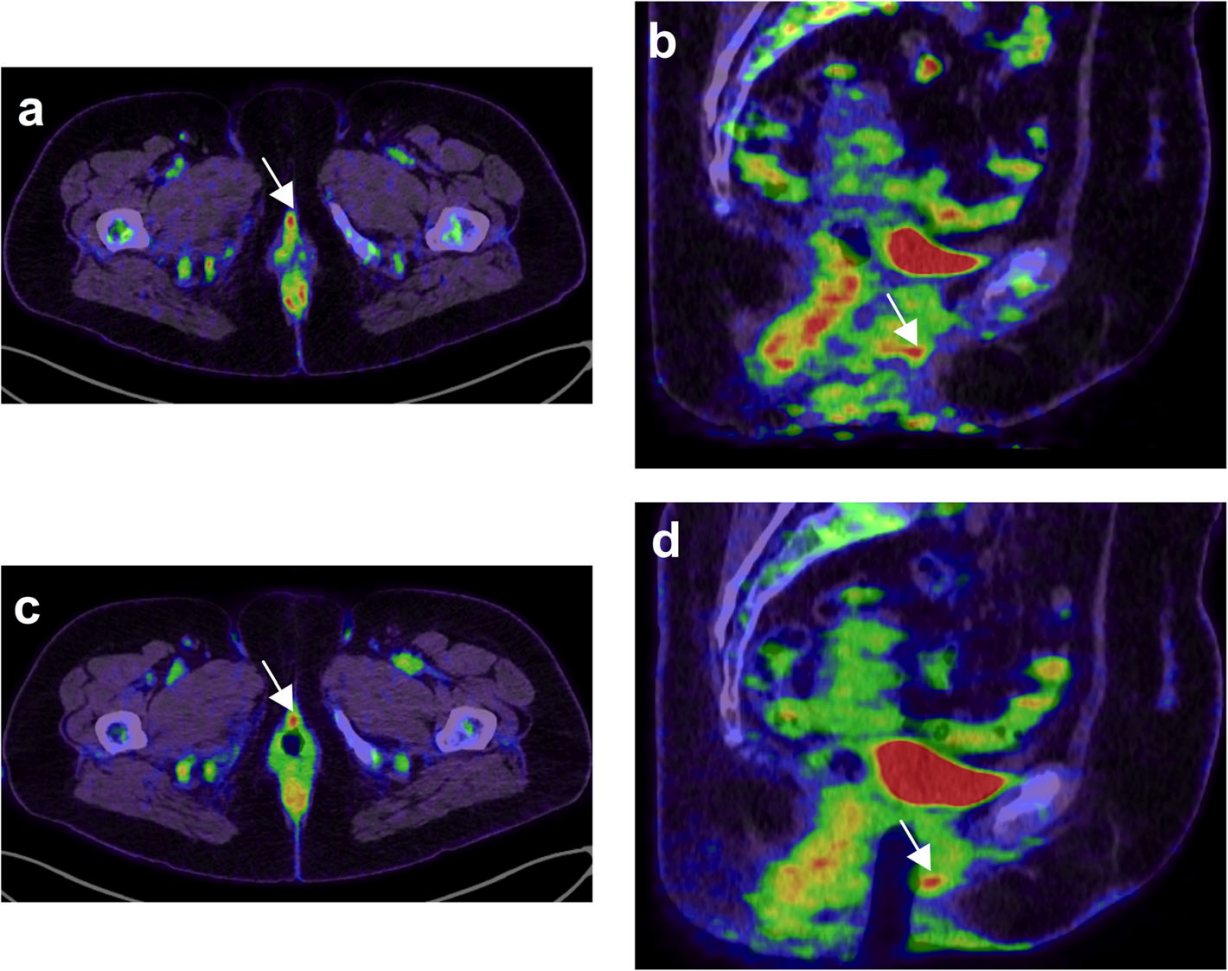
61-year-old woman with suspicion of recurrence for a vulvar melanoma. From left to right, PET/CT fused axial (**a** and **c**) and coronal (**b** and **d**) slices. Patient was scanned before and after introduction of a vaginal tampon because of an indeterminate pelvic focus (white arrow). The images after introduction of the vaginal tampon allowed precise localization of the FDG focus on the anterior vaginal wall

Of note, the vaginal tampon should be inserted properly, as it has been shown by Burger et al. that tampons inserted below the pubococcygeal line can lead to contamination by urinary radiotracer [28]. As for other pelvic tumours, forced diuresis can be considered in addition to the use of a vaginal tampon [24]. This is particularly recommended if there is concern regarding extension to involve the bladder and again, use of radiographic contrast may also help in this situation.

## Withholding metformin Prior to FDG PET/CT (Fig. 8)

In patients receiving metformin, high bowel activity may hamper interpretation of FDG PET images, either by mimicking pathological uptake or by masking lesions located within or near the gut. This may compromise evaluation of primary colorectal cancer or detection of incidental colonic neoplasia, which is not an uncommon benefit of FDG PET/CT [29]. In the context of increasing use of immunotherapy, the ability to diagnose immune-related colitis using FDG PET/CT would also be impaired. This is important since this can be a life-threatening complication and can be identified in advance of symptoms or radiological signs [30] .

**Fig. 8:**
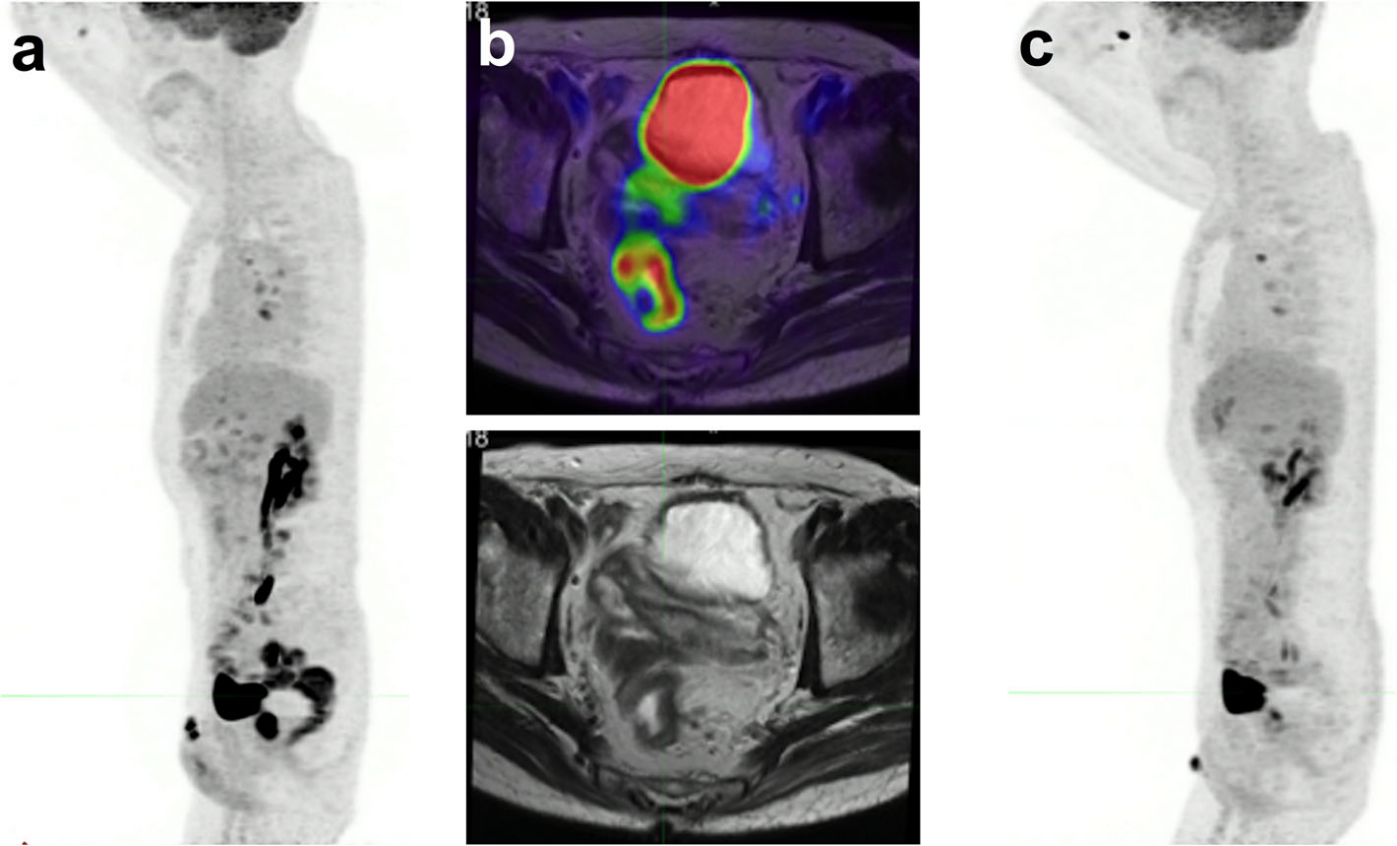
Example of metformin effect. At baseline (**a**) metformin obscured uptake in a known rectal cancer. The baseline uptake in the primary tumour is demonstrated on the fused PET/MRI (**b**) but adjacent uptake in normal bowel compromised assessment of disease extent. The post-treatment scan (**c**) done with metformin withdrawal for 48 h demonstrated a complete metabolic response, which would otherwise have been impossible to discern

Metformin decreases glucose transport from food to plasma and enhances glucose consumption by enterocytes. It is known from preclinical studies that metformin-induced increased uptake selectively involves the colon wall without any significant contamination of the bowel content.

Several studies have evaluated the discontinuation of metformin for periods of 1 to 3 days, with conflicting results. While Oh and colleagues [31] found a 48H withdrawal period to be sufficient, Lee et al. [32] found that a discontinuation time of less than 72H to be suboptimal.

In a prospective randomized single centre trial comparing the effects of a 24H- and 48H metformin discontinuation, Hamidizadeh and colleagues [33] provided solid evidence to support a 48H metformin discontinuation prior to FDG PET scan, with limited impact on glycaemia.

## Suppression of Normal cortical uptake to enhance brain tumour definition (Fig. 9)

High FDG uptake in the normal cerebral cortex and basal ganglia decreases the ability to detect and assess the extent of primary and secondary brain tumours. Although F-18 fluoroethyl-tyrosine (FET) PET/CT overcomes this limitation, when FDG is also required for systemic staging, suppressing cortical uptake of FDG can be advantageous. In the paediatric population, general anaesthesia can markedly reduce uptake in the brain, which may also be desirable from the perspective of minimizing radiation exposure to the developing neurological system but must be carefully balanced against the risks of anaesthesia itself. In adult, use of conscious sedation with midazolam or benzodiazepines may be an alternative. These should be avoided in patients with risk of airway obstruction. The degree of suppression of cortical activity with these interventions is relatively limited and the combination of MRI and FET PET/CT are preferred methods for evaluation of brain lesions.

**Fig. 9:**
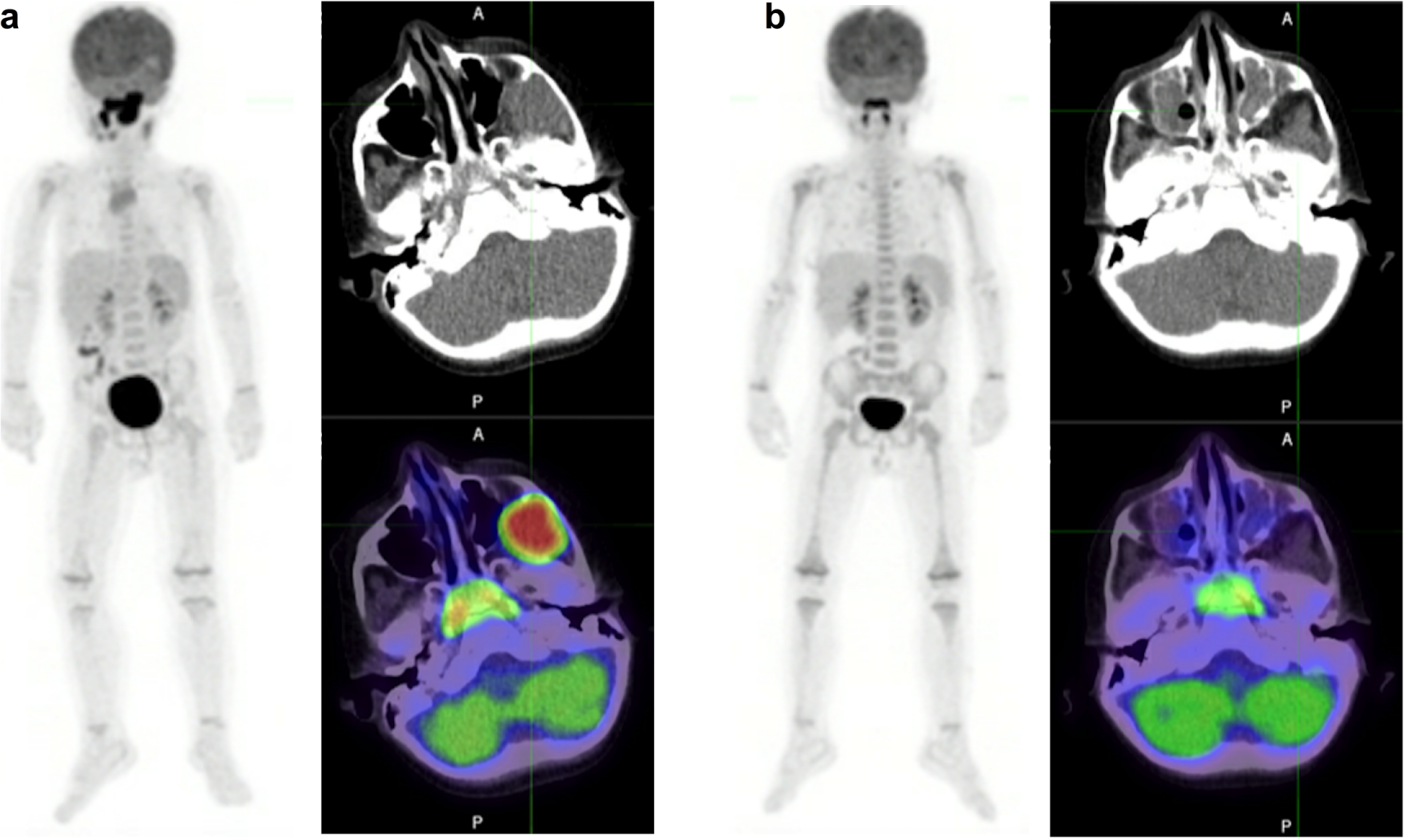
Rhabdomyosarcoma on the left pterygoid fossa in a 6 year-old child treated with chemotherapy demonstrating the marked suppression of cortical brain uptake associated with general anaesthesia (**a**). Note that the same technique was applied for the therapy response scan (**b**). From left to right, MIP body PET CT and PET/CT fused axial slices

## Pain control

Movement is probably the most common cause of degraded image quality and is exacerbated by patient discomfort. This is particularly a problem in patients suffering from cancer. Attention to patient comfort is paramount to achieve high-quality PET/CT studies but judicious use of pain modifying medication should also be considered. It must, however, be recognized that these medications can increase the risk of falls when getting on or off the scanner and careful supervision of patient transfers is required. It is noteworthy that patients in pain may also benefit from fast or even ultra-fast PET imaging, which is achievable on modern PET/CT [34].

## Diagnostic contrast-enhanced CT versus low-dose non-contrast CT

The methodology used for the correlative CT component of PET/CT studies remains one of the more contentious debates in cancer imaging. The incremental radiation dose and risk of contrast reactions associated with diagnostic protocols may be offset by the possibility of improving diagnostic certainty in certain circumstances but, in others, these potential adverse effects serve to add nothing beyond the localising information that can be provided by a low-dose, non-contrast CT. Unfortunately, sometimes the debate has been driven by professional rivalries and not by evidence or even by rational argument. Some PET/CT services are provided entirely within radiology departments and routinely perform full diagnostic CT whereas in other jurisdictions, nuclear medicine physicians never use other than low-dose CT, primarily for attenuation correction purposes [35]. We take the view that all aspects of the acquisition protocol should be optimised to provide the greatest complementary information while minimising risks to the patient. In this context, the acquisition protocols that are optimal for stand-alone CT are not necessarily needed or best for when combined with PET [36]. The CT urogram protocol described above is an example of modifying acquisition protocols to provide complementary diagnostic information.

A further area of controversy is whether or not to include the brain when acquiring oncological PET/CT studies. An argument against doing so is that it potentially provides a false sense of security that brain metastases have been excluded and that by not including the brain, that oncologists are compelled to do the more appropriate investigations of either MRI or contrast-enhanced CT. However, a counterargument is that incidental metastases can be detected, either as hypermetabolic lesions or due to hypometabolism related to vasogenic oedema. Incidental detection of features indicating neurodegenerative or cerebrovascular disease may also impact treatment choices. With tracers that have low or no significant uptake in the brain, inclusion of the head can detect incidental abnormalities that might also provide complementary information to formal neuroimaging [37].

## Conclusion

While slightly increasing the complexity or time investment in performing PET/CT scans, the pharmacological and radiological interventions described above can help imaging specialists to optimize diagnostic information with limited impact on throughput in busy PET units and generally much less impact than having to repeat a non-diagnostic scan or perform another investigation to clarify equivocal findings. Amongst these interventions, management of the diabetic patients and avoidance of BAT uptake are the most frequently encountered issues in PET units and their management is now well documented. When using PET for therapy monitoring purposes, forced diuresis and gastric distension protocols should ideally be kept identical [38] between baseline and interim PET. Although imaging specialists infrequently prescribe medications, knowledge of the contraindications and precautions to be taken is required and, if uncertain, consultation with a specialist clinician is recommended.

## Data Availability

Not applicable.

## Abbreviations

FDG: ^18^F-fluorodeoxyglucose
PET/CT: Positron Emission Tomography with Computed Tomography PET/CT
DM: Diabetes mellitus
BGL: Blood glucose level
BAT: Brown adipose tissue
HIT: Heparin-induced thrombocytopenia
TCC: Transitional cell carcinoma
PSMA: Prostate-specific membrane antigen
FET: ^18^F-fluoroethyl-tyrosine
